# Design and Dynamic Analysis of a Novel Biomimetic Robotics Hip Joint

**DOI:** 10.1155/2015/145040

**Published:** 2015-04-02

**Authors:** Bingyan Cui, Liwen Chen, Zhijun Wang, Yuanhao Zhao, Zhanxian Li, Zhenlin Jin

**Affiliations:** ^1^College of Mechanical Engineering, Hebei United University, Tangshan 063009, China; ^2^College of Mechanical Engineering, Yanshan University, Qinhuangdao 066009, China

## Abstract

In order to increase the workspace and the carrying capacity of biomimetic robotics hip joint, a novel biomimetic robotics hip joint was developed. The biomimetic robotics hip joint is mainly composed of a moving platform, frame, and 3-RRR orthogonal spherical parallel mechanism branched chains, and has the characteristics of compact structure, large bearing capacity, high positioning accuracy, and good controllability. The functions of the biomimetic robotics hip joint are introduced, such as the technical parameters, the structure and the driving mode. The biomimetic robotics hip joint model of the robot is established, the kinematics equation is described, and then the dynamics are analyzed and simulated with ADAMS software. The proposed analysis methodology can be provided a theoretical base for biomimetic robotics hip joint of the servo motor selection and structural design. The designed hip joint can be applied in serial and parallel robots or any other mechanisms.

## 1. Introduction

Biomimetic robotics is the perfect combination of various application purposes bionics technology and robotics fields [1–3]. From the perspective of the robot, the biomimetic robotics is the highest stage in the development of robots. The biomimetic robotics can be to imitate the external shape of organism in nature [4, 5]. The biomimetic robotics joint has bionic joint structure features. By studying the structure of the human body, the design of bionic joint is considered from structural and functional similarity [6, 7].

Biomimetic robotics joint has two kinds of movement form, one is a rotary joint, such as shoulder, elbow, hip, and ankle; these joints can achieve rotatable movement of robotic arm or leg; the other is mobile joints, such as the arm, forearm, and thigh; these mobile joint mechanisms can guide the robot arm or leg movements. In recent years, many domestic and foreign scholars on bionic rotary joint are studied, such as professor Jin et al. [8–10] having proposed a novel 2-DOF elbow joint and analyzed the statics properties, and a novel waist joint has described based on three branched unequal spaced distribution spherical parallel manipulator. Snedeker et al. [11] have analyzed ankle biomechanics.

The hip joint is the most typical and perfect ball and socket joint. At present, the performance analysis and structure design are focus in the research field of the bionic joint. In 2003, Nakamura et al. [12] have designed 6-DOF hip joint based on dual spherical mechanism manufactured of prototype. Saikko [13] promoted an anatomic hip joint simulator which intends to simulate the actual working conditions of the human hip joint. Cheng et al. [14] promoted a novel parallel hip joint simulator based on 3SPS-1PS parallel mechanism, the bionic parallel test platform realized approximately representing the human hip joint motion and provided more reliable experimental data.

Dynamic analysis is an important part of the robot dynamic characteristics and provides the theory basis of dynamic control and servomotor selection [15–17]. Gregorio and Parenti-Castelli [18] have described the mechanism of parallel robot wrist by use of Lagrange method. Zhaocai et al. [19] have analyzed the moving platform inertial parameters on dynamics of flexible planar 3-DOF parallel mechanism affecting the situation. Lagrangian method is a typical dynamic modeling approach based on the system of kinetic energy and potential energy, the Lagrangian operator and partial differential equations of the generalized coordinate variables are established, and the equations are displayed as a matrix form, and using numerical methods for solving partial differential, each force of hip joint dynamics model can be obtained.

From the point of view of bionics, this paper has presented a 3-DOF biomimetic robotics hip joint belonging on structural bionic. The hip joint is mainly for structure design of the body's joints and design goal from three aspects to consider. First of all, the hip joint should have large work space, high bearing capacity, good symmetry, and flexible installation. Secondly, the hip joint should have good structure to facilitate drive installation. Thirdly, hip joint has broad application space and can be used as humanoid robot, walking robot, rehabilitation robot, and so on. In this paper, the biomimetic robot hip joint structural is designed, and kinematics and dynamics are analyzed.

## 2. The Design of the Biomimetic Robotics Hip Joint

Human hip joint [20] is composed of acetabulum, femoral head, femoral neck, ligament, and so on. Form of movement for the human hip is flexion/extension movement in the sagittal plane, adduction/abduction movement in coronal plane, and internal rotation motion/external rotation motion within the cross-sectional plane. The biomimetic robotics hip joint has wide range of motion and flexibility and can achieve three rotate movement. So the design of biomimetic robotics hip joint is mainly considered of hip movement in three coordinate planes. A novel biomimetic robotics hip joint virtual prototype is designed based on 3-RRR orthogonal spherical parallel structure. Technical parameters of biomimetic robotics hip joint are shown in Table 1.

**Figure 1:**
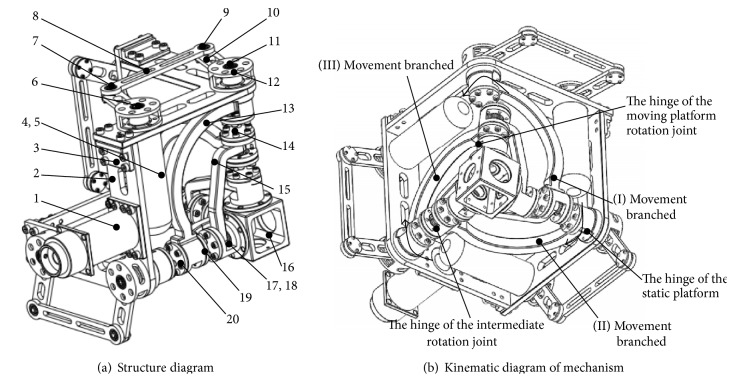
A novel biomimetic robotics hip joint virtual prototype. 1: trunk stent, 2: frame, 3: connectors A, 4: motor, 5: coupling, 6, 7, 9, 11, 14, and 17: revolute joint, 8: long rod, 10: short-bar, 12: connectors B, 13: frame connecting rod, 5: connecting rod, 16: moving platform, 18: rotation bracket, 19: end cover, and 20: screw.

Mechanical design of biomimetic robotics hip joint is shown in Figure 1(a). The driving mechanism is installed on the frame, through the four-bar mechanism to drive the rotation of the hip joint. The biomimetic robotics hip joint mainly contains a moving platform, frame, and three RRR branched chains. The biomimetic robotics hip joint has adopted four-bar mechanism as a transmission mechanism which can enhance the transmission performance, and servomotor has installed on the frame. Compared with industrial series robot joint direct driving scheme, the hip joint has the advantage of the design effectively reducing the axial size of joints, compact structure, reducing the hip joint under the low speed movement of inertia, and making it have better flexible movement.

The hip joint transmission mechanism is composed of a series of parallelograms; the connection order is described and the forward short rob and the back short rob are hinged by the long rod. One end of forward short rod is fixedly connected through the coupling, and the other end of forward short rod is connected to long rod. One end of back short rod is connected to the frame connecting rod. The frame connecting rod is connected to the down connecting rod by a rotating shaft and the moving platform to the down connecting rod by rotating joints. The frame connects the servomotor by screw. Three servomotor rotation axes are perpendicular to each other, the intermediate lever rotation axes are perpendicular, and the axes of the rotating hinges in moving platform are perpendicular too. Hip joint movement is shown in Figure 1(b). There are three identical structure movement branched. Each branch chain motion is: the motor drives the parallel four bar mechanism movement, parallel four bar mechanism through the static platform hinge drives the frame connecting rod, the frame connecting rod through the middle hinge drives the connecting rod movement and through the moving platform hinge drives the motion of the moving platform. Hip joint can be realized its rotation through the interaction of the three branch chains.

## 3. The Kinematic Analysis of Biomimetic Robotics Hip Joint

**Figure 2:**
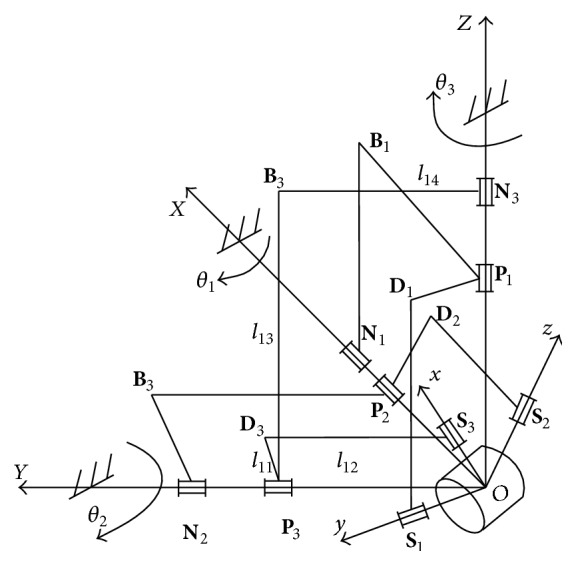
Structure of the hip joint.

The manipulator is design based on 3RRR orthogonal spherical parallel mechanism and is illustrated in Figure 2. For the purpose of analysis, two Cartesian coordinates, the base coordinate frame {**D**} : *O*-*XYZ* and the moving platform coordinate frame {**M**} : *O*-*xyz* are established. Each of the kinematical chains has three revolute joints, where *ON*_*i*_, *OP*_*i*_, and *OS*_*i*_ (*i* = 1,2, 3) represent shaft direction of revolute joints, respectively, and *n*_*i*_, *p*_*i*_, and *s*_*i*_ denote unit vectors of axes (*i* = 1,2, 3), respectively, *ON*_*i*_ of rotating radius is *r*_*N*_, *OP*_*i*_ of rotating radius is *r*_*p*_, *OS*_*i*_ of rotating radius is *r*_*s*_, and the intersection point *O* is rotation center of the manipulators.

According to the structural characteristics of the manipulators and vectors **P**_*i*_ of intermediate shaft relative to the base coordinate frame {**D**} : *O*-*xyz*, **P**_*i*_ is written as

(1) $$\begin{array}{c} P_{i}=\left[\begin{array}{ccc} P_{1} & P_{2} & P_{3} \end{array}\right]=\left[\begin{array}{ccc} 0 & \cos\theta_{2} & -\sin\theta_{3}\\ -\sin\theta_{1} & 0 & -\cos\theta_{3}\\ \cos\theta_{1} & -\sin\theta_{2} & 0 \end{array}\right], \end{array}$$

where *θ*_*i*_  (*i* = 1,2, 3) is input angle.

The direction vector **N**_*i*_ of the frame rotating pair axis on a fixed coordinate system can be written as

(2) $$\begin{array}{c} N_{i}=\left[\begin{array}{ccc} N_{1} & N_{2} & N_{3} \end{array}\right]=\left[\begin{array}{ccc} 1 & 0 & 0\\ 0 & 1 & 0\\ 0 & 0 & 1 \end{array}\right]. \end{array}$$

According to the structure figure of hip joint, the direction cosine of the moving platform rotating pair axis in fixed coordinate system is described as

(3) $$\begin{array}{c} S_{i}=Rs_{i}\;\left(i=1,2,3\right), \end{array}$$

where **R** is rotation matrix,

(4) $$\begin{array}{c} R=R\left(Z,\alpha \right)R\left(Y,\beta \right)R\left(X,\gamma \right)=\left[\begin{array}{ccc} c\alpha c\beta & c\alpha s\beta s\gamma -s\alpha c\gamma & c\alpha s\beta c\gamma +s\alpha s\gamma \\ s\alpha c\beta & s\alpha s\beta s\gamma +c\alpha c\gamma & s\alpha s\beta c\gamma -c\alpha s\gamma \\ -s\beta & c\beta s\gamma & c\beta c\gamma \end{array}\right] \end{array}$$

where *c* is expressed as cos⁡ and *s* is expressed as sin.

**s**_*i*_ is unit vector, and $s_{i}=\left[\begin{array}{ccc} s_{1} & s_{2} & s_{3} \end{array}\right]={\left[\begin{array}{ccc} 0 & 0 & 1\\ 1 & 0 & 0\\ 0 & 1 & 0 \end{array}\right]}^{T}$.

According to (3), vector **S**_*i*_ is written as

(5) $$\begin{array}{c} S_{1}={\left[\begin{array}{ccc} c\alpha s\beta s\gamma -s\alpha c\gamma & s\alpha s\beta s\gamma +c\alpha c\gamma & c\beta s\gamma \end{array}\right]}^{T}\\ S_{2}={\left[\begin{array}{ccc} c\alpha s\beta c\gamma +s\alpha s\gamma & s\alpha s\beta c\gamma -c\alpha s\gamma & c\beta c\gamma \end{array}\right]}^{T}\\ S_{3}={\left[\begin{array}{ccc} -c\alpha c\beta & -s\alpha c\beta & s\beta \end{array}\right]}^{T}. \end{array}$$

For each of the kinematic chains, there is a constraint relation between middle hinge **P**_*i*_ and hinge **S**_*i*_ in moving platform, and the formula can be written as

(6) $$\begin{array}{c} f\left(\theta ,q\right)=P_{i}\cdot S_{i}=0\;\left(i=1,2,3\right), \end{array}$$

where *θ*_*i*_ is input angle, $\theta ={\left(\begin{array}{ccc} \theta_{1} & \theta_{2} & \theta_{3} \end{array}\right)}^{T}$, **q** is output angle and also called generalized coordinates, and $q={\left(\begin{array}{ccc} \alpha & \beta & \gamma \end{array}\right)}^{T}$.

Using the theory of differential, the hip joint kinematic equation are calculated by difference the position vector equation (6), it is written as

(7) $$\begin{array}{c} \dot{q}=-{\left[\frac{\partial f}{\partial q}\right]}^{-1}\left[\frac{\partial f}{\partial \theta }\right]\dot{\theta }=-B^{-1}A\dot{\theta }=J\dot{\theta }, \end{array}$$

where $\dot{\theta }$ is input angle velocity, $\dot{\theta }={\left(\begin{array}{ccc} {\dot{\theta }}_{1} & {\dot{\theta }}_{2} & {\dot{\theta }}_{3} \end{array}\right)}^{T}$, $\dot{q}$ is output angle velocity and also called generalized velocity, $\dot{q}={\left(\begin{array}{ccc} \dot{\alpha } & \dot{\beta } & \dot{\gamma } \end{array}\right)}^{T}$, **J** is Jacobian Matrix of hip joint, and **J** = −**B** ^−1^ **A**. Consider

(8) $$\begin{array}{c} A=\left[\begin{array}{ccc} \left(s\alpha s\beta c\gamma -c\alpha s\gamma \right)s\theta_{1}-c\beta c\gamma c\theta_{1} & 0 & 0\\ 0 & c\alpha c\beta c\theta_{2}+\left(c\alpha s\beta c\gamma +s\alpha s\gamma \right)s\theta_{2} & 0\\ 0 & 0 & s\alpha c\beta s\theta_{3}-c\alpha c\beta c\theta_{3} \end{array}\right],\\ B=\left[\begin{array}{ccc} s\theta_{1}c\gamma s\alpha -s\theta s\beta s\gamma c\alpha_{1} & -s\theta_{1}s\alpha s\gamma c\beta -c\theta_{1}s\gamma s\beta & s\theta_{1}c\alpha s\gamma -s\theta_{1}s\alpha \beta c\gamma +c\theta_{1}c\alpha c\beta c\gamma \\ c\theta_{2}s\beta c\gamma s\alpha -c\theta_{2}s\gamma c\alpha & -s\theta_{2}s\beta c\gamma -c\theta_{2}c\alpha c\beta c\gamma & -s\theta_{2}c\beta s\gamma -c\theta_{2}s\alpha c\gamma +c\theta_{2}s\beta s\lambda \\ -s\theta_{3}s\alpha \beta -c\theta_{3}c\alpha c\beta & -s\theta_{3}c\alpha s\beta +c\theta_{3}s\alpha s\beta & 0 \end{array}\right]. \end{array}$$

## 4. Dynamic Analysis of Hip Joint

Assuming the connecting rods of the hip joint branched chain are uniform density and regular shape, in order to improve machining precision and assembly requirements, the structural sizes of three branched chains are identical, while ignoring the influence of friction.

According to the Lagrangian function, *L* is the difference of the system the total kinetic energy *T* and the total potential energy *Q*. *L* is written as

(9) $$\begin{array}{c} L=T-Q. \end{array}$$

By using the second Lagrange equation [21], the system dynamics equation of hip joint are established as

(10) $$\begin{array}{c} \frac{\partial }{\partial t}\left(\frac{\partial L}{\partial \dot{q}}\right)-\frac{\partial L}{\partial q}=F, \end{array}$$

where **q** is generalized coordinates, $q={\left(\begin{array}{ccc} \alpha & \beta & \gamma \end{array}\right)}^{T}$, $\dot{q}$ is generalized velocity, and $\dot{q}={\left(\dot{\alpha },\dot{\beta },\dot{\gamma }\right)}^{T}$. *F* is generalized output force.

Based on system dynamics equation (10), the kinetic energy and the potential and the corresponding generalized force of each component of the hip joint are calculated.

### 4.1. The Potential Energy of the Hip Joint and Its Corresponding Generalized Output Force

Provided the density of the hip joint bar is *ρ*, the centroid vector of the frame connecting rod is **r**_*i*1_, and the quality is *m*_*j*_; the centroid vector of connecting rod is **r**_*i*2_, and the quality is *m*_*i*_.

**r**_*i*2_ is written as

(11) $$\begin{array}{c} r_{i1}=\left[\begin{array}{ccc} P_{i} & S_{i} & \frac{P_{i}\times N_{i}}{\left|P_{i}\times N_{i}\right|} \end{array}\right]{\left[\begin{array}{ccc} x_{1} & y_{1} & 0 \end{array}\right]}^{T}=x_{1}P_{i}+y_{1}N_{i},\\ r_{i2}=\left[\begin{array}{ccc} P_{i} & S_{i} & \frac{P_{i}\times S_{i}}{\left|P_{i}\times S_{i}\right|} \end{array}\right]{\left[\begin{array}{ccc} x_{2} & y_{2} & 0 \end{array}\right]}^{T}=x_{1}^{\prime }P_{i}+y_{1}^{\prime }S_{i}, \end{array}$$

where the centroid vector coordinate values of the frame connecting rod in plane *N*_*i*_ *B*_*i*_ *P*_*i*_ are *x*_1_ and *y*_1_, and the centroid vector coordinate values of the connecting rod in plane *P*_*i*_ *D*_*i*_ *S*_*i*_ are *x*′ and *y*_1_′.

#### 4.1.1. The Potential Energy of the Frame Connecting Rod and Its Corresponding Generalized Output Force

The potential energy of the frame connecting rod is defined as

(12) $$\begin{array}{c} Q_{j}=m_{j}g\left(\overset{3}{\underset{i=1}{\sum }}r_{i1z}\right). \end{array}$$

Potential energy generalized output force of the frame connecting rod is deduced as

(13) $$\begin{array}{c} \frac{\partial }{\partial t}\left(\frac{\partial Q_{qj}}{\partial \dot{\alpha }}\right)=\frac{\partial }{\partial t}\left(\frac{\partial Q_{qj}}{\partial \dot{\beta }}\right)=\frac{\partial }{\partial t}\left(\frac{\partial Q_{qj}}{\partial \dot{\gamma }}\right)=0. \end{array}$$

According to (13), potential energy generalized output force is obtained

(14) $$\begin{array}{c} F_{qj}=\frac{\partial Q_{qj}}{\partial q}={\left(\begin{array}{ccc} F_{\alpha qj} & F_{\beta qj} & F_{\gamma qj} \end{array}\right)}^{T}=-m_{j}g\left(\begin{array}{c} \frac{\partial \left(r_{11z}+r_{21z}+r_{31z}\right)}{\partial \alpha }\\ \frac{\partial \left(r_{11z}+r_{21z}+r_{31z}\right)}{\partial \beta }\\ \frac{\partial \left(r_{11z}+r_{21z}+r_{31z}\right)}{\partial \gamma } \end{array}\right). \end{array}$$

#### 4.1.2. The Potential Energy of the Connecting Rod and Its Corresponding Generalized Output Force

The potential energy of the connecting rod is defined as

(15) $$\begin{array}{c} Q_{ql}=m_{l}g\left(\overset{3}{\underset{i=1}{\sum }}r_{i2z}\right). \end{array}$$

Potential energy generalized output force of the frame connecting rod is deduced as

(16) $$\begin{array}{c} \frac{\partial }{\partial t}\left(\frac{\partial Q_{ql}}{\partial \dot{\alpha }}\right)=\frac{\partial }{\partial t}\left(\frac{\partial Q_{ql}}{\partial \dot{\beta }}\right)=\frac{\partial }{\partial t}\left(\frac{\partial Q_{ql}}{\partial \dot{\gamma }}\right)=0. \end{array}$$

According to (16), potential energy generalized output force of the connecting rod is obtained as

(17) $$\begin{array}{c} F_{ql}=\frac{\partial Q_{ql}}{\partial q}={\left(\begin{array}{ccc} F_{\alpha ql} & F_{\beta ql} & F_{\gamma ql} \end{array}\right)}^{T}=-m_{l}g\left(\begin{array}{c} \frac{\partial \left(r_{12z}+r_{22z}+r_{33z}\right)}{\partial \alpha }\\ \frac{\partial \left(r_{12z}+r_{22z}+r_{33z}\right)}{\partial \beta }\\ \frac{\partial \left(r_{12z}+r_{22z}+r_{33z}\right)}{\partial \gamma } \end{array}\right). \end{array}$$

#### 4.1.3. The Potential Energy of the Moving Platform and Its Corresponding Generalized Output Force

Provided the structure of moving platform is rules, centroid position of moving platform is located in the center of the moving platform, and the central location of the moving platform and moving coordinate origin is coincident. The centroid vector of the moving platform is **r**_*mz*_, and the quality of moving platform is *m*_*m*_.

The potential energy of the moving platform is defined as

(18) $$\begin{array}{c} Q_{qm}=m_{m}gr_{mz}=0. \end{array}$$

Potential energy generalized output force of the moving platform is deduced as

(19) $$\begin{array}{c} \frac{\partial }{\partial t}\left(\frac{\partial Q_{qm}}{\partial \dot{\alpha }}\right)=\frac{\partial }{\partial t}\left(\frac{\partial Q_{qm}}{\partial \dot{\beta }}\right)=\frac{\partial }{\partial t}\left(\frac{\partial Q_{qm}}{\partial \dot{\gamma }}\right)=0. \end{array}$$

According to (19), potential energy generalized output force of the connecting rod is obtained:

(20) $$\begin{array}{c} F_{qm}=-\frac{\partial Q_{qm}}{\partial q}=0. \end{array}$$

### 4.2. The Kinetic Energy of the Hip Joint and Its Corresponding Generalized Output Force

#### 4.2.1. The Kinetic Energy of the Frame Connecting Rod and Its Corresponding Generalized Output Force

The kinetic energy of the frame connecting rod is defined as

(21) $$\begin{array}{c} T_{tj}=\frac{1}{2}\omega_{j}^{T}\left(I_{j}\right)\omega_{j}, \end{array}$$

where moment of inertia of the frame connecting rod is *I*_*j*_, **ω**_*j*_ is rotation angular velocity of the frame connecting rod, and $\omega_{j}={\left[\begin{array}{ccc} \omega_{1} & \omega_{2} & \omega_{3} \end{array}\right]}^{T}=J\dot{q}$.

Equation (21) can be written as

(22) $$\begin{array}{c} T_{qj}=\frac{1}{2}\left[\begin{array}{ccc} \dot{\alpha } & \dot{\beta } & \dot{\gamma } \end{array}\right]\left[K_{j}\right]{\left[\begin{array}{ccc} \dot{\alpha } & \dot{\beta } & \dot{\gamma } \end{array}\right]}^{T}, \end{array}$$

where [**K**_*j*_] is matrix and [**K**] = [**J**]^*T*^[**I**_*j*_][**J**].

Kinetic energy generalized output force of the moving platform is deduced as

(23) $$\begin{array}{c} F_{tj}=\frac{\partial T_{tj}}{\partial q}={\left(\begin{array}{ccc} F_{\alpha tj} & F_{\beta tj} & F_{\gamma tj} \end{array}\right)}^{T}=\left[H_{j}\right]\left[\ddot{q}\right]+\left[C_{j}\right]\left[\dot{q}\right], \end{array}$$

where **H**_*j*_ is the inertial moment of the frame connecting rod, **H**_*j*_, [**H**_*j*_] = [**E**][**K**_*j*_]. **C**_*j*_ is the centrifugal and Coriolis forces, $\left[C_{j}\right]=\left[E\right]\left(\partial \left[K_{j}\right]/\partial t\right)-\left(1/2\right){\left[\dot{q}\right]}^{T}\left(\partial \left[K_{j}\right]/\partial q\right)$.

Where **E** is identity matrix.

#### 4.2.2. The Kinetic Energy of the Connecting Rod and Its Corresponding Generalized Output Force

The kinetic energy of the connecting rod is defined as

(24) $$\begin{array}{c} T_{tl}=\frac{1}{2}m_{l}{\left\|v_{li}\right\|}^{2}, \end{array}$$

where centroid velocity of the connecting rod is *v*_*li*_, *v*_*li*_ = *v* + *v*_*e*_ = *r*_*p*_ *ω*_*i*_ + *y*_1_′*ω*_*pi*_; *r*_*p*_ is a distance from the hinge point points *P*_*i*_ to the center of rotation *O*; angular velocity of the motor input is *ω*_*i*_, and angular velocity of the connecting rod is *ω*_*pi*_.

Equation (24) can be written as

(25) $$\begin{array}{c} T_{tl}=\overset{3}{\underset{1}{\sum }}\frac{1}{2}m_{l}{\left(r_{p}\omega_{i}+y_{1}^{\prime }\omega_{pi}\right)}^{2}. \end{array}$$

The kinetic energy of the connecting rod is divided into three parts, the kinetic energy is two, respectively rotate around the center of rotation and the kinetic energy of an implicated motion. Because implicated velocities *v* and *v*_*e*_ are perpendicular to each other, the dot product of implicated velocity *v* and *v*_*e*_ is zero, and the kinetic energy of implicated velocity is given as

(26) $$\begin{array}{c} T_{tle}=0. \end{array}$$

According to (24) to (26), the kinetic energy of the connecting rod is written as

(27) $$\begin{array}{c} T_{tl}=\frac{1}{2}\left[\begin{array}{ccc} \dot{\alpha } & \dot{\beta } & \dot{\gamma } \end{array}\right]\left[K_{l}\right]{\left[\begin{array}{ccc} \dot{\alpha } & \dot{\beta } & \dot{\gamma } \end{array}\right]}^{T}, \end{array}$$

where [**K**_*l*_] is matrix, [**K**_*l*_] is written as [**K**_*l*_] = [**J**][**I**_*l*1_][**J**]^*T*^ + [**K**_*p*_][**I**_*l*2_][**K**_*p*_]^*T*^, and **I**_*l*1_ and **I**_*l*2_ are moment of inertia,

(28) $$\begin{array}{c} K_{p}=\left(\left[E\right]-\frac{r_{p}}{\sqrt{r_{p}^{2}+r_{s}^{2}}}\left[J\right]\right). \end{array}$$

Kinetic energy generalized output force of the connecting rod is deduced as

(29) $$\begin{array}{c} F_{tl}=\frac{\partial T_{tl}}{\partial q}={\left(\begin{array}{ccc} F_{\alpha tl} & F_{\beta tl} & F_{\gamma tl} \end{array}\right)}^{T}=\left[H_{l}\right]\left[\ddot{q}\right]+\left[C_{l}\right]\left[\dot{q}\right], \end{array}$$

where **H**_*l*_ is the inertial moment of the connecting rod and is written as [**H**_*l*_] = [**E**][**K**_*l*_].

The centrifugal and Coriolis forces are **C**_*l*_, $\left[C_{\text{l}}\right]=\left[E\right]\left(\partial \left[K_{l}\right]/\partial t\right)-\left(1/2\right){\left[\dot{q}\right]}^{T}\left(\partial \left[K_{l}\right]/\partial q\right)$.

#### 4.2.3. The Kinetic Energy of the Moving Platform and Its Corresponding Generalized Output Force

The moving platform has rotational motion, and the kinetic energy of the moving platform is established as

(30) $$\begin{array}{c} T_{tm}=\frac{1}{2}\left[\begin{array}{ccc} \dot{\alpha } & \dot{\beta } & \dot{\gamma } \end{array}\right]\left[I_{tm}\right]{\left[\begin{array}{ccc} \dot{\alpha } & \dot{\beta } & \dot{\gamma } \end{array}\right]}^{T}, \end{array}$$

where moment of inertia of the moving platform is **I**_*tm*_.

Kinetic energy generalized output force of the moving platform is deduced as

(31) $$\begin{array}{c} F_{tm}=\frac{\partial }{\partial t}\left(\frac{\partial T_{tm}}{\partial \dot{q}}\right)-\frac{\partial T_{tm}}{\partial q}={\left(\begin{array}{ccc} F_{\alpha tm} & F_{\beta tm} & F_{\gamma tm} \end{array}\right)}^{T}=\left[H_{m}\right]\left[\ddot{q}\right]+\left[C_{m}\right]\left[\dot{q}\right], \end{array}$$

where the inertial moment of the frame connecting rod is **H**_*m*_, [**H**_*m*_] = [**E**][**I**_*tm*_].

The centrifugal and Coriolis forces are **C**_*m*_, $\left[C_{m}\right]=\left[E\right]\left(\partial \left[I_{tm}\right]/\partial t\right)-\left(1/2\right){\left[\dot{q}\right]}^{T}\left(\partial \left[I_{tm}\right]/\partial q\right)$.

### 4.3. The Dynamic Model

According to formulae (10) to (31) into (10), the kinetic model of the hip joint is written as

(32) $$\begin{array}{c} F=F_{qj}+F_{ql}+F_{qm}+F_{tj}+F_{tl}+F_{tm}. \end{array}$$

Static equation of hip joint is established as

(33) $$\begin{array}{c} F=G\tau , \end{array}$$

where **τ** is driver input torque, **G** is force Jacobian matrix, and **G** = (**J** ^−1^)^*T*^.

In summary, if the linear density of error, friction, small changes of abrasion, and other factors is ignored, the dynamic equation of hip joint is described as

(34) $$\begin{array}{c} \tau =J\left(\left[H\right]\left[\ddot{q}\right]+\left[C\right]\left[\dot{q}\right]+F_{q}\right), \end{array}$$

where **F**_*q*_ is gravity term, **F**_*q*_ = **F**_*qj*_ + **F**_*ql*_ + **F**_*qm*_, **H** is symmetric positive definite moment of inertia, [**H**] = [**H**_*j*_] + [**H**_*l*_] + [**H**_*m*_], **C** is the centrifugal and Coriolis force, and [**C**] = [**C**_*j*_] + [**C**_*l*_] + [**C**_*m*_].

Servomotor torque of the hip joint is expressed as

(35) $$\begin{array}{c} \tau =J\left(\left[H\right]\left[\ddot{q}\right]+\left[C\right]\left[\dot{q}\right]+\left[F_{q}\right]\right). \end{array}$$

## 5. Kinematics and Dynamic Calculation and Simulation of Hip Joint

Assume the structure parameters of the biomimetic robotics hip joint are *l*_*i*2_ = *l*_*i*4_ = 110 mm, *l*_*i*1_ = 50 mm, and *l*_*i*3_ = 180 mm and cross-sectional dimension of the connecting rod are *B* = 12 mm, *T* = 10 mm, and *t* = 8 s.

The Solidworks model of hip joint is imported in ADAMS software and the appropriate constraints are added. The quality characteristic parameters of each part of the hip joint model are provided according to the actual material of the physical prototype. Function of displacement variation with time is planned:

(36) $$\begin{array}{c} \alpha =-\frac{\sin\left(2t+\pi \right)}{2}\;\left(\text{rad}\right)\\ \beta =\frac{\sin\left(2t+\pi /2\right)}{2}\;\left(\text{rad}\right)\\ \gamma =-\frac{\sin\left(2t+\pi \right)}{2}\;\left(\text{rad}\right). \end{array}$$

**Figure 3:**
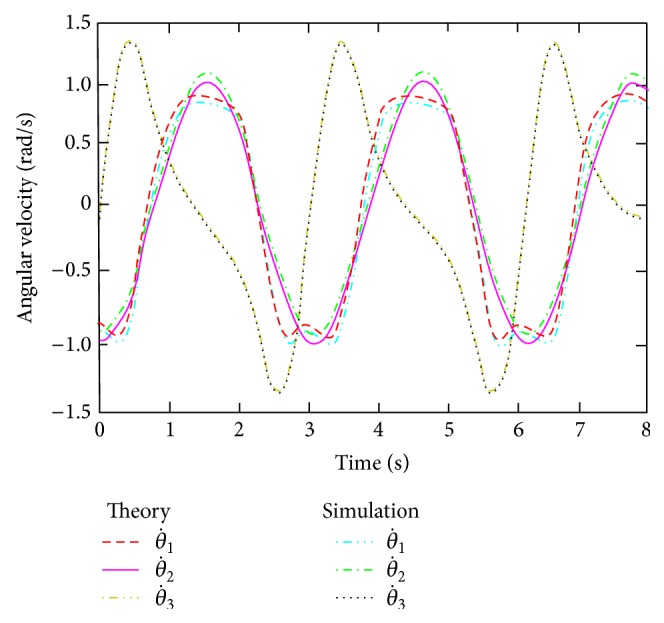
Driving angular velocity curve of servomotor.

According to a given trajectory of the hip joint, through inverse kinematics solution equation (7), variation of the hip joint angular velocity of the drive motor is obtained by inverse kinematics analysis, as shown in Figure 3. The variation of the hip joint angular velocity of the drive motor is obtained by inverse kinematics analysis.

According to the movement rule of the hip joint moving platform, the kinematics of the hip joint are simulated. The angle velocity curve of drive motor is obtained and shown in Figure 3. From Figure 3, it can be seen that the angular velocity simulation curve of the driver motor and theoretical calculation curve is consistent, and the error value is very small.

**Figure 4:**
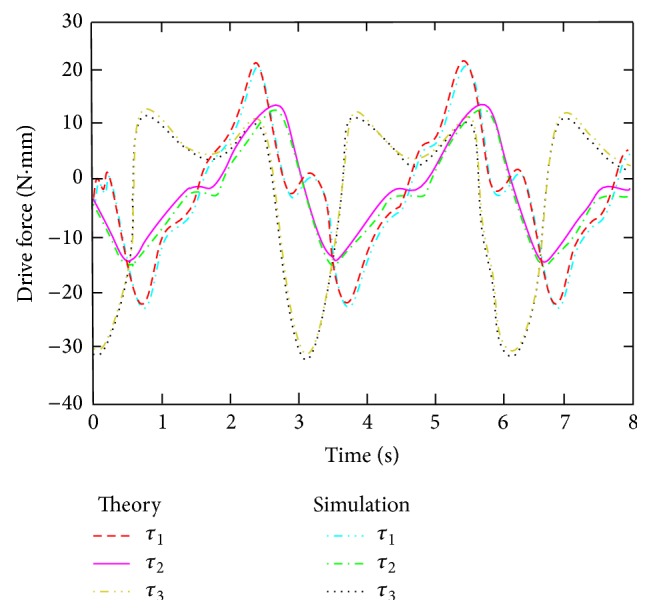
Driving force curve of servomotor.

According to the given trajectory formula of the hip joint, generalized velocity is deduced, given time zone calculation meshing which time interval is *δt*. Hip joint kinetic energy and potential energy to calculate the initial condition is *t* equals 0. Using numerical calculation backward difference method, partial differential equation of generalized force is calculated and the computing program is compiled by Matlab. The variation of the hip joint drive force is obtained through dynamic equation calculated, as shown in Figure 4. Figure 4 shows the maximum and minimum value of driving force and then describes the driving force regularly change with the change of time.

According to the movement rule of the hip joint moving platform, the dynamics of the hip joint are simulated. The driving force of drive motor is obtained and shown in Figure 4. From Figure 4, it can be seen that the driving force simulation curve of the driver motor and theoretical calculation curve almost coincide, and the error value is very small. The maximum drive torque of three motors is 30.15 N·mm. The analysis results prove that the calculation is consistent well with the simulation result.

## 6. Conclusions

A novel biomimetic robotics hip joint has been designed, and virtual prototype is designed. Kinematic equation of hip joint is derived based on position vector method. Using Lagrange method, the dynamic model of the hip joint is established. According to the movement rule of the hip joint moving platform, this paper has gained the driving force and driving angle velocity movement law of the hip joint. The analysis results prove that simulation curve of the driver motor and theoretical calculation curve are consistent and obtained that the maximum driving torque is 30.15 N·mm. This paper provides a theoretical basis for hip joint structural optimization and motor selection and should be useful for the further research and practical application of the biomimetic robotics.

## Figures and Tables

**Table 1:** The technical parameters of the biomimetic robotics hip joint.

Parameter	Technical index
Degree of freedom	3
Flexion/°	130
Extension/°	45
Internal rotation/°	40
External rotation/°	40
Abduction/°	60
Adduction/°	45
Source voltage/V	24
Maximum velocity/(r/min)	30
Weight/N	110
*l*_*i*2_ = *l*_*i*4_/mm	120
*B*/mm	12
*l*_*i*1_/mm	70
*l*_*i*3_	180
Motor	Maxon 218012
Coupling	Maxon 223085
Encoder	MR TL256-1024-CPT

## Terms

***θ***_*i*_: Input angle
**P**_*i*_: Vector of the intermediate shaft in fixed coordinate system
**N**_*i*_: Vector of the frame rotating pair in fixed coordinate system
**S**_*i*_: Vector of the moving rotating pair in fixed coordinate system
**R**: Rotation matrix
$\dot{\theta }$: Input angle velocity
**J**: Jacobian matrix
**q**: Generalized coordinates
$\dot{q}$: Generalized velocity
**F**: Output force
**H**: Real symmetric inertia matrix
**C**: Coriolis inertia force
*I*: Moment of inertia
**E**: Identity matrix
**τ**: Driver input torque
**G**: Force Jacobian matrix
*T*: Kinetic energy
*Q*: Potential energy
*t*: Time
*l*: Length
**r**: The centroid vector of rod
*m*: Quality
*r*: Rotating radius.
